# Individualized tourism recommendation based on self-attention

**DOI:** 10.1371/journal.pone.0272319

**Published:** 2022-08-25

**Authors:** Guangjie Liu, Xin Ma, Jinlong Zhu, Yu Zhang, Danyang Yang, Jianfeng Wang, Yi Wang

**Affiliations:** 1 College of Computer Science and Technology, Changchun Normal University, Changchun, Jilin, China; 2 School of Data and Computer Science, Sun Yat-Sen University, Guangzhou, China; 3 CRRC Changchun Railway Vehicles CO.,LTD, Changchun, Jilin, China; Hanyang University, KOREA, REPUBLIC OF

## Abstract

Although the era of big data has brought convenience to daily life, it has also caused many problems. In the field of scenic tourism, it is increasingly difficult for people to choose the scenic spot that meets their needs from mass information. To provide high-quality services to users, a recommended tourism model is introduced in this paper. On the one hand, the tourism system utilises the users’ historical interactions with different scenic spots to infer their short- and long-term favorites. Among them, the users’ short-term demands are modelled through self-attention mechanism, and the proportion of short- and long-term favorites is calculated using the Euclidean distance. On the other hand, the system models the relationship between multiple scenic spots to strengthen the item relationship and further form the most relevant tourist recommendations.

## Introduction

The system of recommendation is used in all aspects of life. It not only saves time for users when searching for information, it also brings better benefits to service providers [1, 2]. However, there have been few studies on scenic recommendation systems. Li et al. [3] fit the implicit preferences of users through demographic attribute information, obtaining their preferences for different population attributes through a hierarchical sampling of statistical models, and generating recommendation lists from the mined user preferences. Based on an analysis of the information evaluations made by other users, Alexander et al. [4] also recommend the current best attractions for users to attend based on user preferences and the current status of their areas.

Among the many recommendation techniques [5], item-based collaborative filtering (ICF) comes out to be the most widely applied [6, 7] owing to its low data dependency and easy algorithm implementation [8, 9]. The key to ICF is to discover similarities between items, then suggested similar items to user depending on information regarding the historicals they have [10–12]. The similarity is usually determined by the history of user interaction. Despite the prevalence and effectiveness of ICF methods, they are inadequate because of the fact that they allow only coarse-grained, collaborative similarity relationships lacking concrete semantics. He et al. [13] introduced relational collaborative filtering (RCF) which integrates multiple item relationships to form recommendations, but does not consider the existing rich sequential patterns in users’ historical interactions.

In a user-item historical interaction, the associated timestamps are recorded with the passage of time. All such data can reflect the strong correlation and even causality that exists in the user behaviors [14–18]. Koren et al. [16] argued that simulating the time dynamics is key when designing recommendation systems or general customer preference models, after which they proposed models that can track time-changing behavior over the entire data lifecycle. Wu et al. [19] modelled the temporal evolution of ratings using a recurrent neural network(RNN), which were not designed for the recommendation domain. In 2016, YouTube [6] proposed to apply deep learning to video recommendation and achieved extremely good results. Since then, the spread of deep learning techniques has blossomed, resulting in a variety of papers, academic exchanges, and industrial applications in the field of recommender system. He et al. [20] also used a neural network structure to model user-item interaction data, while using multilayer perceptual machines to learn user-item interaction equations. Convolutional neural network (CNN) can find the features information in a large amount of data and can generalized to similar problems with similar type [21–25]. Recurrent neural network perform well in modelling of temporal dynamics [19, 26–28]. Zhang et al. [29] combined the user’s history on websites to make simple recommendations to the user; however, the rich sequential patterns in user interaction and the multiple relationships that exist between items are also extremely important. To better learn the sequence representation and multiple relationships present in the items, a neural sequence recommendation model for scenic spots, i.e., a Self-Attention based individualized Tourism Recommendation (ATTR), is proposed in this paper. The system model the sequence of user interactions through a self-attention and maintain item relationships through an embedding operation. These operations provide accurate analysis of the user’s interests to effectively predict the most suitable item for the user. Finally, prove the usefulness of the model experimentally. The contributions of this work are are listed below:

- This paper proposes a new model for sequential recommendation tasks. The model combines the analysis of users’ long- and short-term favorites with modelling the relationship between items to better infer the following behavior of the target user.
- This paper analyses the interaction data between users and scenic items to get users’ long- and short-term favorites. In this method, short-term preferences are modelled using a self-attention mechanism, and item embedding is enhanced by preserving the relationship structure between scenic items.
- Finally, to validate the methodology introduced in this paper by two datasets, demonstrating that the frame attains the most advanced performance. The remainder of this paper is organised as follows: Section 2 explains the knowledge required for the model and the complete model formation process. Section 3 reports on the experimental and performance analyses of this approach. Finally, Section 4 presents a review of this work.

## Materials and methods

### Attention mechanism

In the process of reading and communicating, our attention is not allocated to every word in a balanced manner. To make computers more adaptable to human communication, they must learn to selectively forget and associate the context, which is a mechanism known as an attention mechanism [30–32]. The attention mechanism has developed as a hot research topic in neural networks and good results has been gained in areas such as computer vision [33], image captioning [34], and machine translation [35], where the original idea of this mechanism lies in the efficient computation of attention distribution. Bahdanau et al. [35] were among the first to use attention as a mechanism to search for relevant parts from the input sequence for the current target item. Sanghyun et al. [36] also proposed adding an attention mechanism to both the feature channel and feature space dimensions of a CNN. The attention mechanism allows the RNN not to be limited by the input sequence length, allowing the CNN to acquire the information that requires more attention. Many more studies in the field of recommendation have been conducted on attentional mechanisms. Li et al. [37] proposed session-based recommendations to generate recommendation results from short sessions. Zhou et al. [38] addressed the problem that a user’s interest in different items should be modelled in a way that does work by weighting the user’s historical behavior sequences with attention.

In this paper, a new concept of self-attention is addressed and compared to standard attention, self-attention concerns the interaction of two sequences, where the attentional weight of one sequence depends on the other. Self-attention is predicated on attention mechanism. With the successful application of attention mechanism by the Google team [39], self-attention mechanism has also rapidly occupied hotspots in various fields due to several advantages such as its fast training speed, and has successfully solved numerous problems.

### Item-based collaborative filtering

ICF is aimed at mining historical user behavior and uses it as a basis for making recommendations [8, 9]. User prediction scores for items are derived from the similarity between items, where the relationship between the items is called collaborative similarity. The most popular among ICFs is FISM [9], which offers a way to better express user information by portraying the user as an expression of the item that the user has liked. Both neural network enhancement methods [8] and involving local potential energy spaces [40, 41] have been the subject of extensive research in this area. Although these improvements have improved the performance of ICF, the coarse granularity of item relationships and the absence of semantic meaning remain a problem, making it difficult to produce better recommendations. The main differences in this work in comparison to existing approaches are the use of self-attention mechanisms and how the item relationship data can be modelled to introduce relational structures between item embeddings.

First, the users’ short-term intention is obtained by modelling their historical interaction behavior by the self-attention, and the influence of long- and short-term intentions on the user is analysed based on the Euclidean distance. Second, item embedding is enhanced by preserving the relational structure between scenic spots.

### Methodology

**Fig 1:**
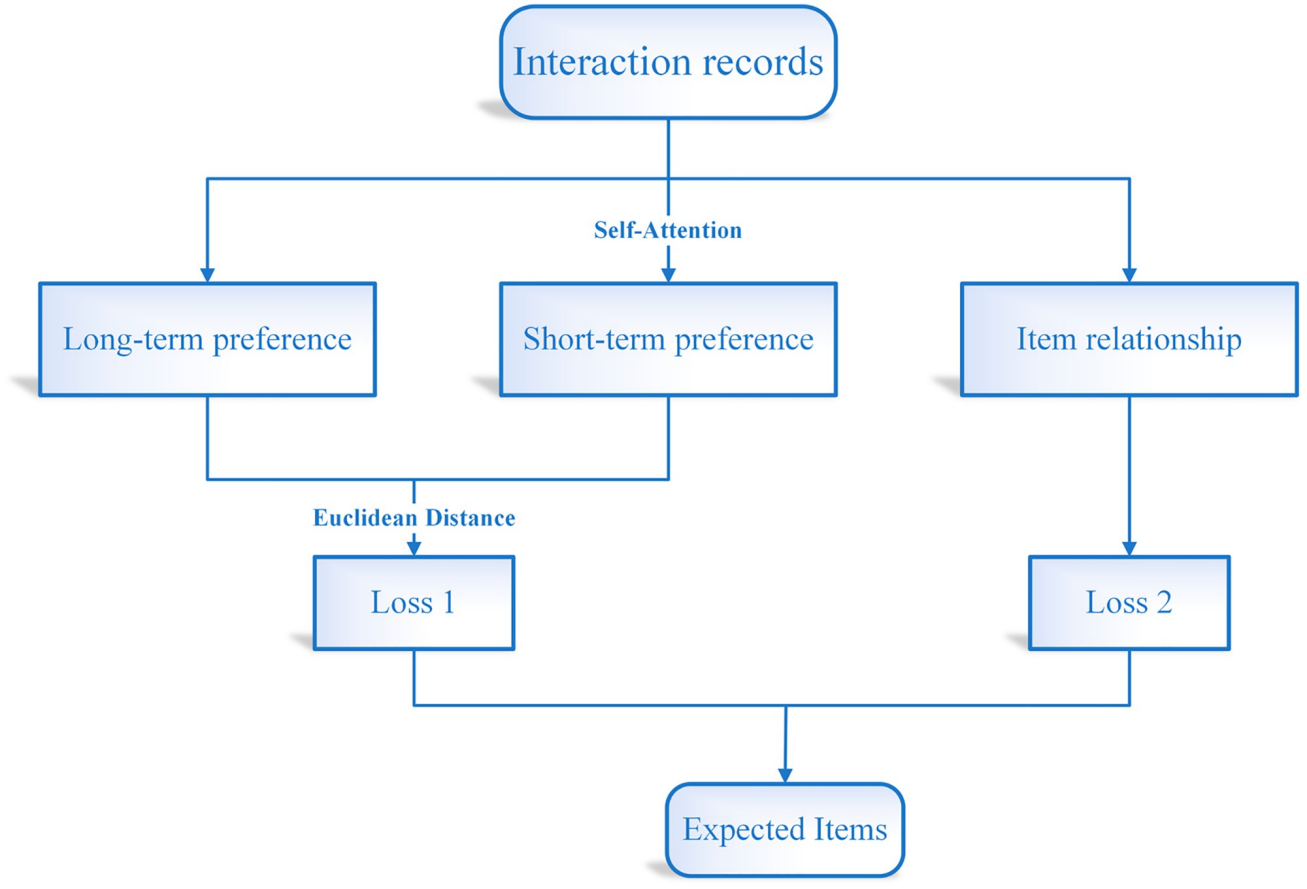
Frame diagram for this paper. The left part of the figure models the user’s long- and short-term intentions, of which the short-term intentions are derived from the self-attention mechanism. The right half of the figure models the item relations.

This section first explains how to get uses’ long- and short-term intentions, and then build upon this by introducing the modelling of the item relationships. Fig 1 shows the framework of the model. The left half in this figure models the user preferences, in which the short-term interests are modelled by self-attention model, the right half of the figure models the item relationships.

This paper assumes that U is the user collections and I is the item collections, where |*U*| = *M* and |*I*| = *N*. Here, $I^{u}=\left(I_{1}^{u},\cdots ,I_{|I^{u}|}^{u}\right)$ represents the items in the chronological user interaction record, where *I*^*u*^ ∈ *I*. Define the relationship between item pair (*i*, *j*) as the set of *r* = < *relation* *type* *t*, *relation* *value* *v* >. Table 1 presents the symbolic representation of this model.

**Table 1:** Symbolic representation of the model in this paper.

Notation	Narrate
*U*	user collections
*I*	item collections
*I* ^*u*^	historical interaction sequence for a user $u:(I_{1}^{u},\cdots ,I_{|I^{u}|}^{u})$
*I*_*r*_(*i*, *j*)	item relation function, when *I*_*r*_(*i*, *j*) = 1 means that relation *r* exists for item *i* and item *j*
T	relation type collections
V	relation value collections
*q*^*i*^ ∈ *R*^*N* × *d*^	embedding of item *i* ∈ *I*
*x*_*t*_ ∈ *R*^*N* × *d*^	embedding of relation type t∈T
*z*_*v*_ ∈ *R*^*N* × *d*^	embedding of relation value v∈V

#### User preference modelling

A better understanding of short-term preferences can be gained by analysing the user’s recent behavior. In this paper, their interaction recordings were modelled through self-attention to obtain short-term intentions. The self-attention is special case where the query, value, and key are identical in attention, and all consist of the interaction data about user and item. The mechanism of attention is essentially a weighted summation of the values of the elements, and the query and key are used to calculate the weighting factors for the corresponding values.

Assume that the user’s recent preference are acquired from the nearest L(e.g.,5,10) item interaction, and item can be expressed as a d-dimensional embedding vector. Set all item embeddings denoted as *X* ∈ *R*^*N* × *d*^. Stack the most recent *L* items sequentially to obtain the matrix, as in Eq (1):

$$\begin{array}{c} X_{t}^{u}=[\begin{array}{cccc} X_{(t-L+1)1} & X_{(t-L+1)2} & \cdots & X_{(t-L+1)d}\\ \vdots & \vdots & \vdots & \\ X_{(t-1)1} & X_{(t-1)2} & \cdots & X_{(t-1)d}\\ X_{t1} & X_{t2} & \cdots & X_{td} \end{array}]. \end{array}$$ (1)

The latest *L* items are a subset of *I*^*u*^. In this part, user *u*’ query, key, and value of at step *t* are equal to $X_{t}^{u}$. First, the query and key are projected into the same space via a nonlinear activation function *ReLU* with shared parameters, as in Eqs (2) and (3):

$$\begin{array}{c} Q^{\prime }=ReLU\left(X_{t}^{u}W_{Q}\right). \end{array}$$ (2)

$$\begin{array}{c} K^{\prime }=ReLU\left(X_{t}^{u}W_{K}\right). \end{array}$$ (3)

here, *W*_*Q*_ ∈ *R*^*d* × *d*^ is the weight matrix of the query and *W*_*K*_ ∈ *R*^*d* × *d*^ is the weight matrix of the key. Then, the product of *Q*^′^ and *K*^′^ are calculated. To avoid overly large results, the result is divided by a scale $\sqrt{d}$, and the affinity matrix is computed as in Eq (4) [39]:

$$\begin{array}{c} s_{t}^{u}=softmax\left(\frac{Q^{\prime }K^{\prime T}}{\sqrt{d}}\right). \end{array}$$ (4)

$s_{t}^{u}$ is an *L* × *L* matrix that shows the similarity between the *L* terms, and *d* is initialized to a larger value (e.g. 100). For avoiding high-patch points between equal query and key vectors, the masked affinity matrix diagonal operation is adopted before softmax is applied.

Then, let the value and $X_{t}^{u}$ be the same. Unlike other cases in which linear transformations are typically used to map values, the use of identity mapping in this model is beneficial. In other application areas such as word embedding, values are usually pre-trained feature embeddings, whereas in this paper, values are composed of parameters that need to be learned. The difficulty of viewing the actual parameters can be made by adding a linear or non-linear transformation. Queries, keys, and values are not sensitive to transformations in the same way because queries and keys are used as subsidiary factors.

Finally, the resulting matrix is multiplied by the value to produce a representation of the weight summation, as in Eq (5):

$$\begin{array}{c} a_{t}^{u}=s_{t}^{u}X_{t}^{u}. \end{array}$$ (5)

The short-term interests of users are represented by this output. To learn individual attention representations, the user’s short-term intention is represented by the minimal embedding in the *L* self-attention denotation, as indicated in Eq (6):

$$\begin{array}{c} m_{t}^{u}=\text{min}\;a_{t}^{u}. \end{array}$$ (6)

The above formulas also operate with the *sum*, *max* and *mean*, the validity of which is compared in a later section. The time signal is not included in the above model and needs to be added to this model to preserve the sequence pattern. In the next work, we propose to provide time signals for the query and key through positional embedding. Next, sinusoidal signals of different frequencies are added to the input using a geometric time-scale sequence. The two sine signals form the time embedding (*TE*), as shown in Eqs (7) and (8):

$$\begin{array}{c} TE\left(t,2i\right)=\text{sin}\left(t/10000^{2i/d}\right). \end{array}$$ (7)

$$\begin{array}{c} TE\left(t,2i+1\right)=\text{cos}\left(t/10000^{2i/d}\right). \end{array}$$ (8)

Here, *t* denotes the time step, *i* denotes the dimension. Before the query and key are nonlinearly transformed, *TE* is added.

After modelling short-term intentions, considering the combination of the users’ general and long-term preferences will yield better overall recommendations for users. As with the latent factor approach, a latent factor is assigned to each user and each item. Set *U*^′^ ∈ *R*^*M* × *d*^ and *V* ∈ *R*^*N* × *d*^ as the potential factors for both the user and item. Affinity between *u* and *i* is measured by the Euclidean distance, as shown in Eq (9) [42]:

$$\begin{array}{c} \left|\right|U_{u}^{\prime }-V_{i}{\left|\right|}_{2}^{2}. \end{array}$$ (9)

If user *u* likes item *i*, then this distance should be small, and if user *u* does not like item *i*, then this distance should be large.

Predict the items (denoted by $I_{t+1}^{u}$) which user u are probably interacts with at time step *t* + 1 by modelling their short- and long-term preferences at the previous *t* steps. For consistency, Euclidean distance is used to predict the weight of short- and long-term preferences, which is used as a recommendation score, as indicated in Eq (10):

$$\begin{array}{c} y_{t+1}^{u}=\omega ||U_{u}^{\prime }-V_{I_{t+1}^{u}}{\left|\right|}_{2}^{2}+\left(1-\omega \right)||m_{t}^{u}X_{t+1}^{u}{\left|\right|}_{2}^{2}. \end{array}$$ (10)

In the formula, the first term is the product of the control factor *ω* and the user *u* long-term intention score for the next item $I_{t+1}^{u}$, and the next term is the product of the control factor *ω* and the user *u* short-term interest score for item $I_{t+1}^{u}$. Here, *V* and *X* are distinct parameters, whereas both $V_{H_{t+1}^{u}}$ and $X_{t+1}^{u}$ represent the *t*+ 1th item of the embedding vector.

This work aims to predict not just one item, but the next few items of user *u*. This requires our model to capture the jumping behavior. Make *T*^+^ indicate the next *T* items that interact with the user in the groundtruth. It is also necessary to collate items which user do not interact with and denote them with *T*^−^. *T*^+^ and *T*^−^ from set *I*. The goal of doing this procedure is to easily learning the model variables using pairwise sorting, using the losses as in Eq (11):

$$\begin{array}{c} L_{1}=\sum_{\left(u,i\right)\in T^{+}}\sum_{\left(u,i\right)\in T^{-}}{\left[y_{i}^{u}+\gamma -y_{i}^{u}\right]}_{+}+{\lambda ||\theta ||}_{2}^{2}. \end{array}$$ (11)

here, *θ* = {*X*, *V*, *U*, *W*_*Q*_, *W*_*K*_} stands for the parameters of the model, and *γ* represents the margin that divides *T*^+^ and *T*^−^.

#### Item relational data modeling

**Fig 2:**
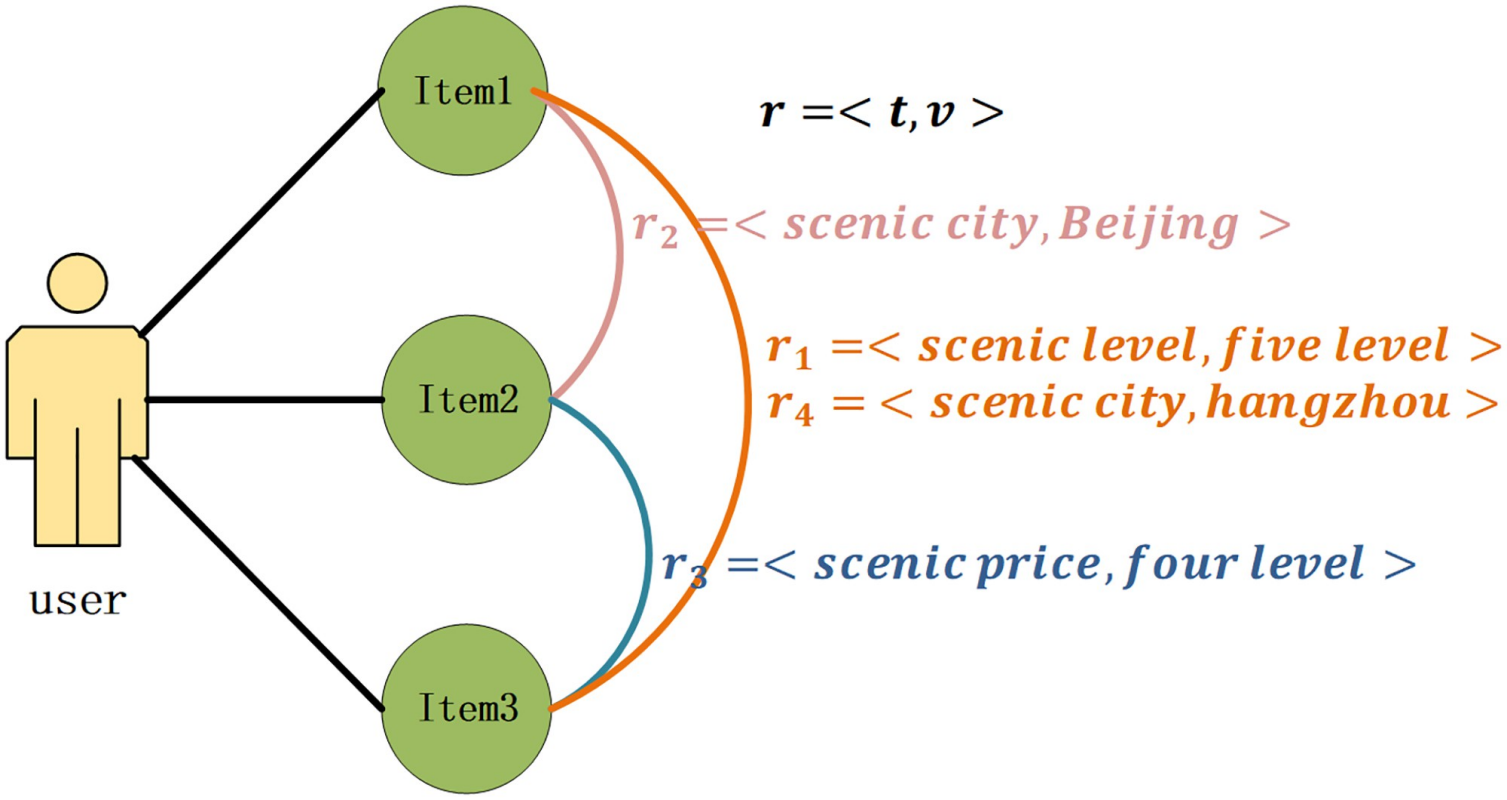
Diagram illustrating the multiple relationships between items in the user interaction history. Each relation *r* is described by type *t* and value *v*. There is a relationship *r*_2_ between Item 1 and Item 2, the relationship type is the scenic city, and the relationship value is Beijing. Multiple relations may also exist between two items.

The following is the modelling of the item relationships. Fig 2 shows an example of multiple relationships among items from the user interaction data. The item relationship *r* as a function of relation type *t* and relation value *v*, *r* = < *t*, *v* >. For example, there is a relationship *r*_2_ between Item1 and Item 2, and Item 2 and Item 3 also exist relationship *r*_3_. There may be more than one relationship between two items, as shown in Fig 2, there are two relationships between Item 1 and Item 3. Knowledge graphs, as an emerging type of auxiliary information, have gradually captured the eye of society over the past few years. The knowledge graph store real-world entities and the relationships between entities whose nodes indicate entities or concepts and whose edges indicate all kinds of contextual relationships between entities or concepts. A knowledge graph consists of several ternary groups (h,r,t), where h and t stand the head and tail nodes of a relationship, and *r* represents the relationship. An effective way to derive signals in relational data is to embed knowledge graphs into a space of continuous vectors. However, the direct use of knowledge graph embedding techniques has certain problems in the recommendation domain:

1. Item relationships are defined as a two-level structure: relationship type and relationship value. To represent this relationship correctly, the relationship between the two levels of model fidelity must be considered. Therefore, single embeddings cannot be assigned to item relationships. To resolve this problem, it use the two levels of layered parts as relational embedding, which can be expressed using Eq 12:
  $$\begin{array}{c} r=x_{t}+z_{v}. \end{array}$$ (12)
2. Unlike traditional knowledge graphs that are represented by directed graphs, item relationships are invertible (i.e. relation *r* is valid for (h,t) and (t,h)), form an undirected graph structure. The most widely used graph, TransE [18], maps the relationship between two entities to an embedded action between them, whereby h+r≈t when (h,r,t) holds, where the embedding of the head entity is denoted by h, the embedding of the relation is denoted by *r*, and the embedding of the tail entity is denoted by t. Comprised of the above, TransE frames the triplet’s scoring function as $f\left(h,r,t\right)=||h+r-{t||}_{2}$, where∥⋅∥_2_ denotes the *L*_2_ criterion of the tensor. Owing to the non-directional structure, both h+r≈t and t+r≈h are obtained. An objective function optimized in this way may yield an insignificant solution with *r* ≈ 0 and h≈t. To solve this problem, the origin is found to be the subtractive operation of TransE, which applies only to directed structures. We require a model that solves the exchange rule (i.e.f(h,r,t)=f(t,r,h)). DistMult [43] is another advanced approach for knowledge graph embedding, and expresses the rating function as $f\left(h,r,t\right)=h^{T}M_{r}t$, where *M*_*r*_ is a matrix denotation of *r*. DistMult clearly fulfils these requirements. From the above, we define items *i* and *j* with relation *r* as (*i*, *r*, *j*), and their function as Eq 13:
  $$\begin{array}{c} f\left(i,r,j\right)=q_{i}^{T}\cdot diag\left(r\right)\cdot q_{j}. \end{array}$$ (13)

where *diag*(*r*) represents diagonal matrix with the same value of diagonal elements and *r*. In this part, it is necessary to maximize *f*(*i*, *r*, *j*) with positive instances and minimize it with negative instances. The target function is refined by comparing the points of the interacted triplets (*i*, *r*, *j*) to the uninteracted triple (*i*, *r*, *j*^−^), as shown in Eq 14:

$$\begin{array}{c} L_{2}=-\sum_{\left(i,r,j,j^{-}\right)\in D_{R}}\text{ln}\;\sigma \left(f\left(i,r,j\right)-f\left(i,r,j^{-}\right)\right). \end{array}$$ (14)

here *D*_*R*_ is defined as Eq 15:

$$\begin{array}{c} D_{R}=\left\{\left(i,r,j,j^{-}\right)|i,j,j^{-}\in I\wedge I_{r}\left(i,j\right)=1\wedge I_{r}\left(i,j^{-}\right)=0\right\}. \end{array}$$ (15)

#### Model learning

During the recommendation phase, the item’s recommendation score is calculated, and the candidate items are sorted in ascending order. Then the user is recommended the highest ranked items. To efficiently learn the recommendation parameters and retain the relational structure between project embeddings, the sequential recommendation section and the relational modelling section are learned end-to-end using a multitasking framework. The overall target function of this work is given by Eq 16:

$$\begin{array}{c} L=L_{1}+L_{2}. \end{array}$$ (16)

**Fig 3:**
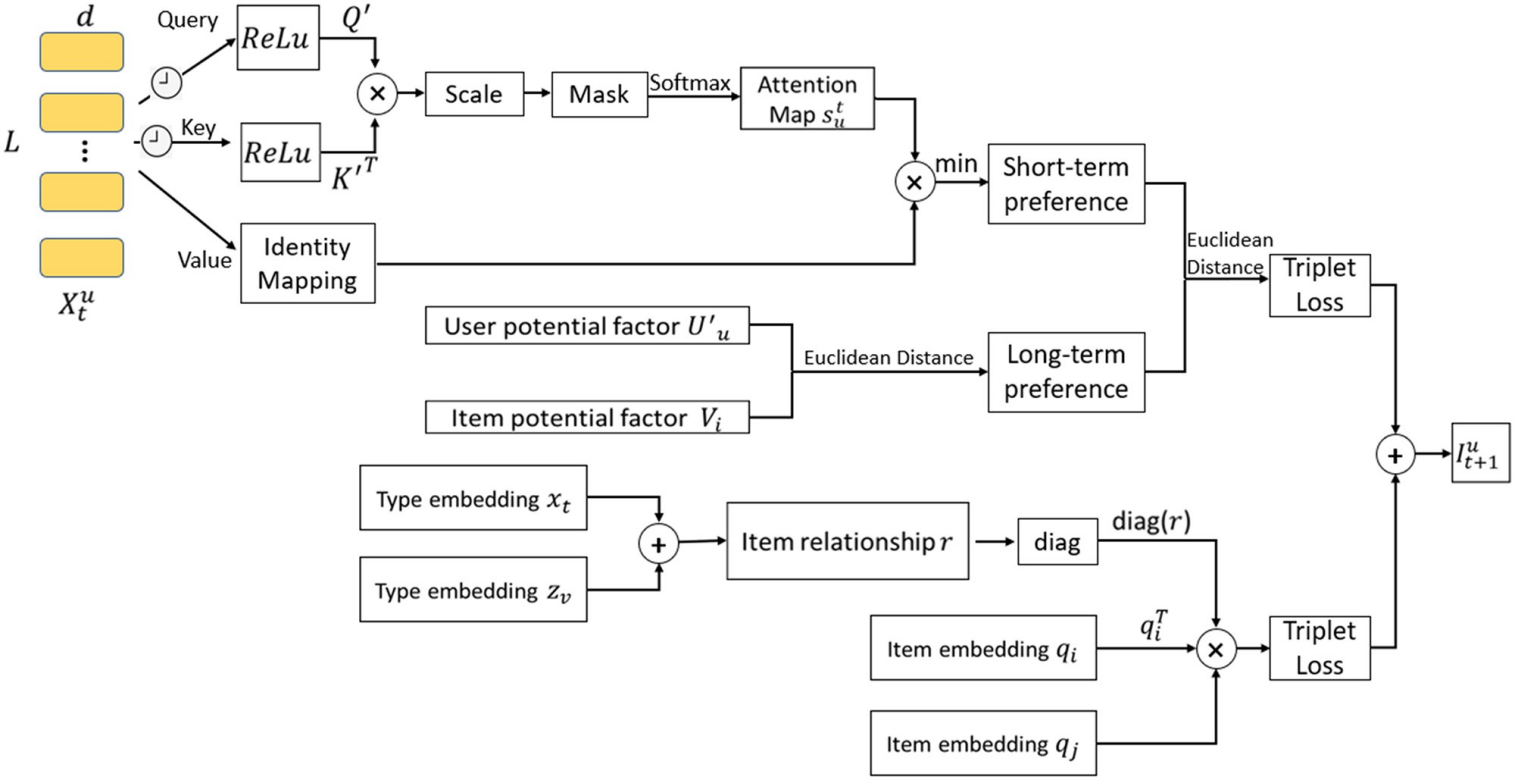
Structural diagram of this paper. The upper half of the diagram is composed of modelling the users’ long- and short-term intentions and the lower half is the modelling of the item relationships. The self-attention mechanism is adopted to analyse the users’ short-term intentions, and the Euclidean distance is applied to simulate the influence of long- and short-term intentions.

Fig 3 shows the structure of the paper. It contains the long- and short-term intentions of the user, and it contains the relationships between items. Both were added together to form the eventual recommendation list. The short-term intentions of users are inferred through self-focused networks, as well as by building the entire system within the framework of measurement learning.

## Results and discussion

In this part, two real datasets were used to experiment and assess the proposed sequence recommendation model. The aim of this work is by answering the following issues:

**RQ1**: Does the self-attention based model introduced in this paper perform the advanced performance?
**RQ2**: What are the implications of the critical hyper-parameters?

### Dataset descriptions

In this paper, two datasets were used for the experiments: Tourism Dataset, and the hetrec2011 dataset on movie recommendations.

1. **Tourism Website(https://github.com/DATASU10/DATASET)**
  Tourism dataset is a dataset built on the basis of the “2018 Cloud Mobile Cup Scenic Spot Word-of-Mouth Score Prediction”(Comes from the National Tourism Big Data Challenge organized by Yunnan University and Yunnan Provincial Society of Applied Statistics, and the official competition platform is DataFountain) and the “Tourist scenic spots Data in the ModelWhale Community” (https://www.heywhale.com/mw/dataset/6108b262911b330017451cc7/file), and they are both publicly available. Tourism dataset contains 70,544 data records from 850 users for 678 scenic spots and is available at https://github.com/DATASU10/DATASET. The dataset contains two parts: the interaction records of users and scenic spots (including userID, scenic spotID, ratings, and timestamps), and the relationship data of 678 scenic spots (including scenic spotID, scenic spot city, scenic spot level, and scenic spot ticket price). The interaction records between users and scenic spots are composed of the “2018 Cloud Mobile Cup Scenic Spot Word-of-Mouth Score Prediction”, and the relationship data of scenic spots are composed of the “Tourist scenic spots data in the ModelWhale Community”.
2. **Hetrec2011(https://grouplens.org/datasets/hetrec-2011/)**
  Hetrec2011 is a public dataset available at https://grouplens.org/datasets/hetrec-2011/. It contains 199997 data records, which is a dataset for recording interactions between users and movies. Select data on the interaction record between users and movies (including userID, itemID, ratings, and timestamps) and data on the relationship between movies (itemID, country of movie, movie genre, and movie director). The main parts used are the four files user_ratedmovies-timestamps.dat, movie_genres.dat, movie_director.dat and movie_countries.dat.

Datasets with explicit scores are converted into implicit feedback. The detailed statistics for the dataset are presented in Table 2.

**Table 2:** Dataset description.

Dataset	Tourism Website	Hetrec2011
#user	678	511
#item	850	8708
#interaction	70544	199997

### Evaluation metrics

For each user, this paper use nearest item for testing and conducts hyper-parameter tuning using the second nearest item. The hit rate (*HR*), mean reciprocal (*MRR*) and normalized discounted cumulative gain (*NDCG*) were taken to evaluate the capability of all models. The HR measures the correctness of the recommendation. The HR is reported to have a stop value of k (k = 5, 10), which is defined as Eq 17:

$$\begin{array}{c} HR@k=\frac{1}{M}\sum_{u\in U}1\left(R_{u,g_{u}}\leq k\right). \end{array}$$ (17)

Here, *g*_*u*_ is the rank produced by the model for this groundtruth item.

The mean reciprocal rank indicates where the model ranks the item. *MRR*@*k* allocates better marks to items on the recommended list. The *MRR* is defined as Eq 18:

$$\begin{array}{c} MRR@k=\frac{1}{M}\sum_{u\in U}\frac{1}{R_{u,g_{u}}}. \end{array}$$ (18)

Here, $R_{u,g_{u}}$ is the rank for the ground truth item.

*NDCG*@*k* places highly relevant items at the top of the recommendation list, emphasizing the sequential nature of the items. The *NDCG* is defined as Eq 19:

$$\begin{array}{c} NDCG@k=\sum_{i=1}^{k}\frac{2^{r_{i}}-1}{{\text{log}}_{2}\left(i+1\right)} \end{array}$$ (19)

Here, *r*_*i*_ is the user’s preference value for the *i*-th item among the first *k* items.

### Compared models

The model introduced in this paper is compared with traditional approaches and more advanced models. Specifically, the introduced model is measured against the baseline below:

**AttRec** [29]: This is a sequence-aware recommendation model that uses a self-attention mechanism to model the interaction between the user and history, resulting in a final user representation.
**TiSASRec** [44]: The approach proposes a time interval self-attention mechanism to model the time interval in user interactions to better infer user preferences.
**LSSA** [45]: The method proposes a multilayer long- and short-term self-attention network for sequential recommendation that combines long-term and short-term favorites of users to capture their complex preferences.
**RCF** [13]: This approach proposes a new item-based collaborative filtering framework designed to integrate relationships across multiple items for better recommendations.
**FISM** [9]: This is the most advanced ICF model that describes users in terms of the average aggregation of interaction item embeddings.
**NAIS** [8]: This method enhances the FISM by displacing the average aggregation of the FISM with an attention-based summation.
**MF** [46]: It uses the inner product of the user and the interaction item to simulate the user preferences. This is a standard matrix factorization method.

Given that the adaptive gradient optimizer is adopted in this model, the learning rate is fixed at 0.05. To assure a balanced comparison of the model performance, the latent dimension *d* for the model introduced in this paper and for all models in which this variable is present is fixed at 100. The effect of *d* in this model is explained in the following section. The regularization rate λ is adjusted between 0.1, 0.01, 0.001, 0.0001. The dropout rate is adjusted between 0, 0.3, 0.5, 0.7. The weight factor *ω* is adjusted between 0, 0.2, 0.4, 0.6, 0.8, 1.0. The sequence length of *L* is fixed at 5. The target length *T* is fixed at 3. The margin *γ* of the hinge loss is set to 0.5 for all datasets. The experimental part of this paper is realized in Python using TensorFlow.

### Model comparison

Table 3 shows the experimental outputs for the seven baselines and the model introduced in this paper on two datasets. The table shows that this model always achieves a good performance on both datasets. This also establishes the validity of all methods applied.

**Table 3:** Comparison of hit rate (HR), NDCG and MRR performance of all models on two datasets.

Models	Metirc	AttRec	RCF	TiSASRec	LSSA	FISM	NAIS	MF	ATTR
**Tourism Website**	HR@5	0.056	0.057	0.061	0.060	0.038	0.061	0.069	**0.074**
	NDCG@5	0.044	0.038	0.037	0.036	0.025	0.037	0.040	**0.054**
	MRR	0.049	0.031	0.026	0.030	0.021	0.043	0.031	**0.053**
	HR@10	0.110	0.103	0.120	0.050	0.056	0.124	0.104	**0.127**
	NDCG@10	0.045	0.052	0.053	0.032	0.031	0.058	0.051	**0.064**
	MRR	0.047	0.037	0.040	0.027	0.023	0.038	0.035	**0.052**
**Hetrec2011**	HR@5	0.019	0.005	0.024	0.029	0.003	0.001	0.001	**0.032**
	NDCG@5	0.012	0.002	0.015	0.013	0.002	0.007	0.003	**0.021**
	MRR	0.016	0.001	0.020	0.018	0.001	0.005	0.006	**0.022**
	HR@10	0.035	0.009	0.034	0.037	0.007	0.025	0.009	**0.041**
	NDCG@10	0.018	0.004	0.022	0.023	0.003	0.011	0.004	**0.026**
	MRR	0.018	0.002	0.021	0.021	0.002	0.007	0.001	**0.023**

**Fig 4:**
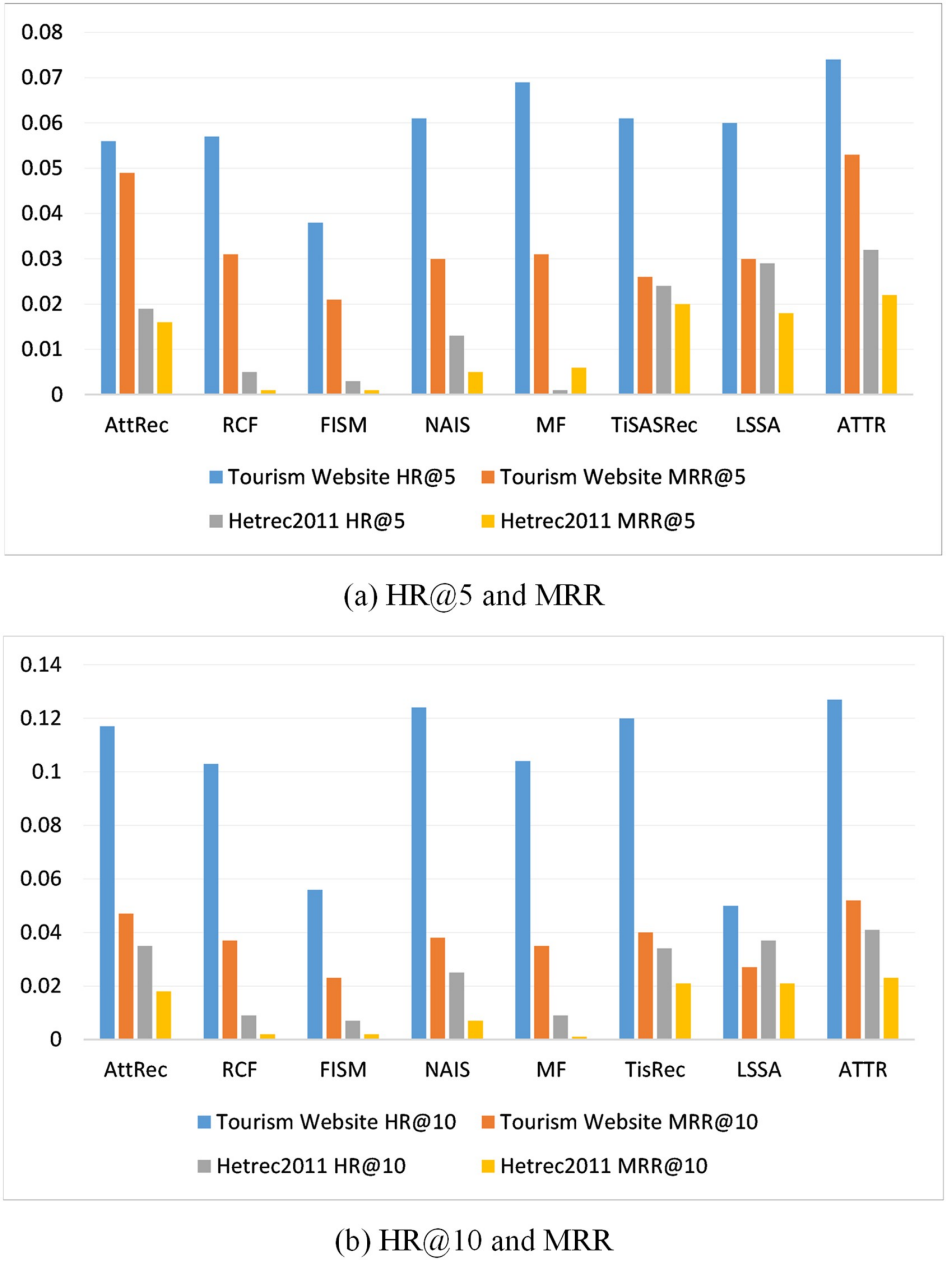
Histograms comparing the model with other baselines in the case of the top-*k*(*k*=5,10) recommendations, respectively. From the figure, it is clear that the model introduced in this paper achieves the best performance on both datasets.

In contrast to the sequence-aware recommendation model, AttRec, TiSASRec and LSSA does not model the relationships between items, but only the historical interactions of users using the self-attention mechanism. The importance of modelling the item relationships can also be illustrated from this perspective. Compared to RCF, FISM, and NAIS, the latter only considers the collaborative similarity. In this work not only the long and short term preferences of users are modeled, but also the item relationships are analyzed, where the self-attention mechanism is used to model the short term preferences of users and the Euclidean distance is used to calculate the respective shares of long and short term preferences, in addition to considering sequential modeling, which is the main reason for the improvement. From this perspective, the results demonstrate the importance of the sequential modelling. Fig 4(a) and 4(b) illustrate histograms comparing the model introduced in this paper with other baselines in the case of the top-*k*(*k*=5,10) recommendations, respectively. The graph shows that the model introduced in this work results in the highest performance on both datasets. In summary, the model presents in this paper largely outperforms all baselines, which clearly answers **RQ1**.

### Parametric analysis

In this section, the model is analyzed in depth and, designed to better recognize the actions of our model in response to **RQ2**.

**Effect of aggregation approach.** The representation of the user’s short-term intent is obtained using four types of aggregation. The usability of these four aggregation methods is then discussed. Table 4 illustrates the results of the four different aggregation methods, with *HR*@*k* and *MRR*(k = 5,10) as the metric. It can be seen that “minimum” achieves satisfactory results for both datasets.

**Table 4:** HR@k and MRR(k = 5,10) of this model with different aggregation methods on two dataset(Tourism Website and Hetrec2011).

	Tourism Website				Hetrec2011			
	HR@5	MRR@5	HR@10	MRR@10	HR@5	MRR@5	HR@10	MRR@10
*Min*	**0.074**	**0.053**	**0.127**	**0.052**	**0.032**	**0.022**	**0.041**	**0.023**
*Mean*	0.065	0.053	0.118	0.051	0.025	0.018	0.037	0.019
*Sum*	0.034	0.028	0.096	0.033	0.023	0.015	0.035	0.020
*Max*	0.067	0.047	0.122	0.050	0.025	0.015	0.037	0.018

**Fig 5:**
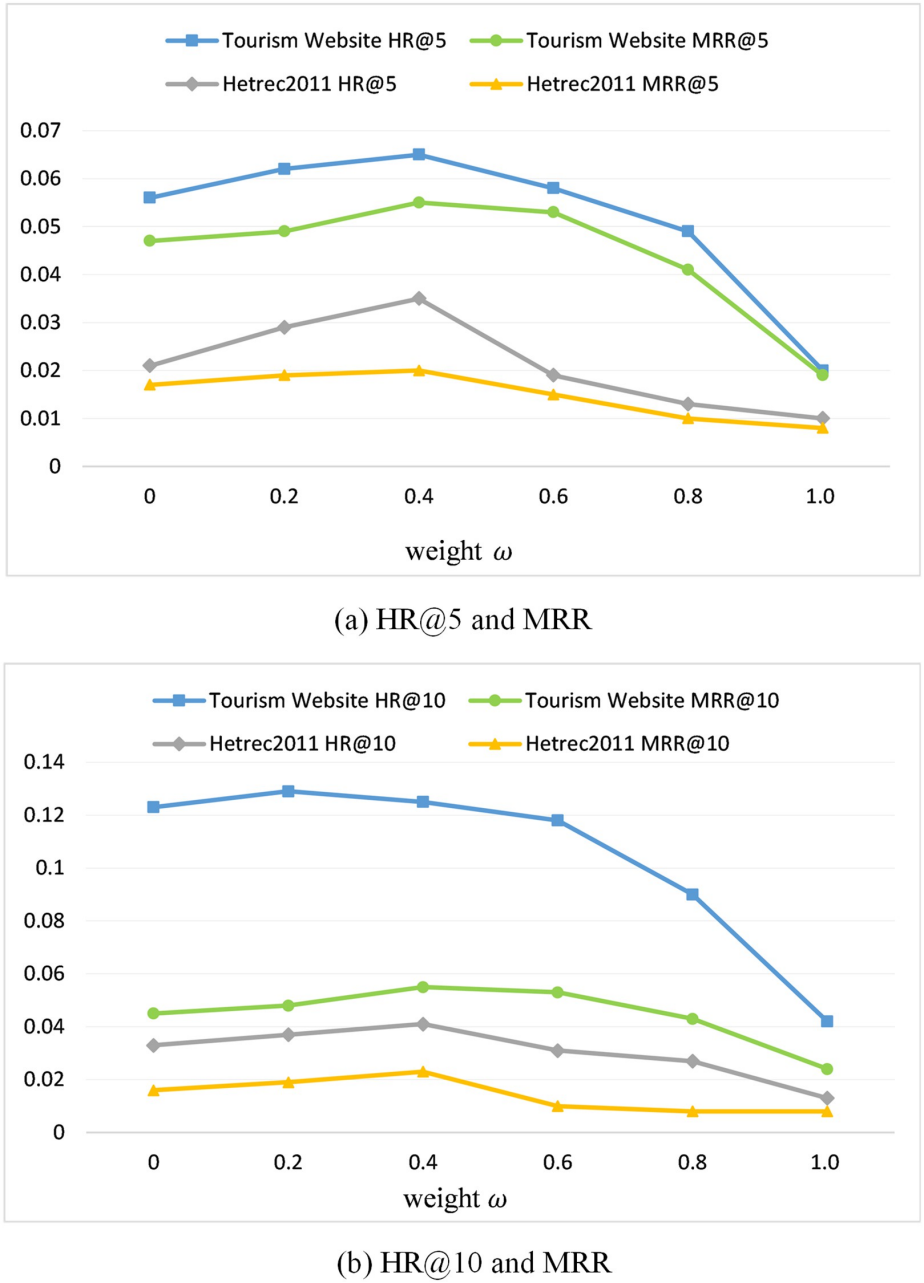
Effects of the weight *ω*.

**Effect of weight *ω*.** Fig 5(a) and 5(b) illustrates the results of setting different parameters *ω* on the two datasets with *HR*@*k* and *MRR*(*k*=5,10) as measures. Parameter *ω* manages the effects of the model for both the short- and long-term effects. From Fig 5, it is desirable to set the value of *ω* at between 0.2 and 0.4, indicating that short-term intent is more important to a sequence recommendation.

**Fig 6:**
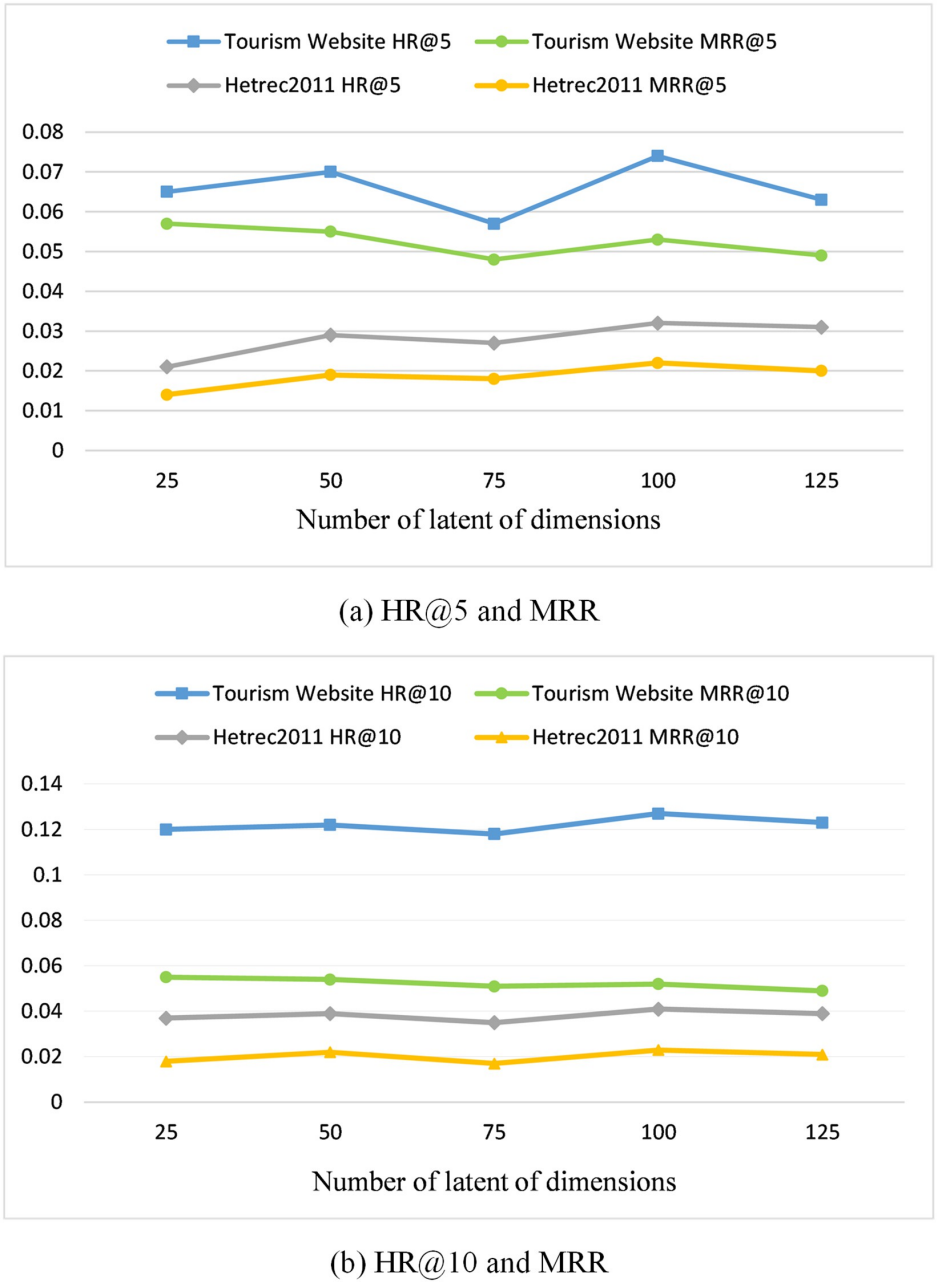
Effects of number of latent dimensions *d*.

**Effect of the number of dimensions *d*.** Fig 6(a) and 6(b) shows the results for different numbers of dimensions *d* on two datasets using *HR*@*k* and *MRR*(*k*=5,10) as a measure and keeping the other parameters the same. From the figure, it can be concluded that a larger dimensionality does not mean a higher performance, considering the overfitting problem.

**Fig 7:**
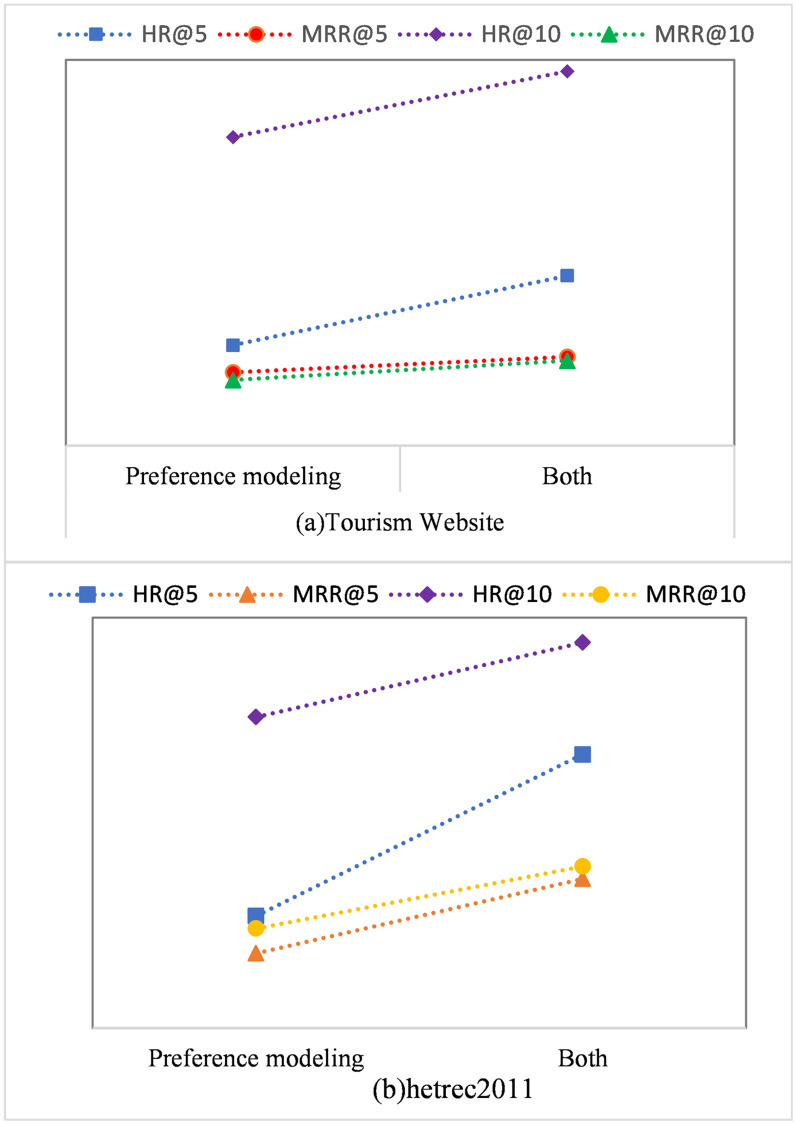
The effects of item relationship modelling on two different datasets. *MRR*@*k* is the *MRR* that predicts the next *k* items.

**Effect of modelling the item relationships.** Fig 7(a) and 7(b) show the impact of item relationship modelling on the model presented in this paper on two different datasets. It can be concluded from the figure that the model introduced in this paper is more effective than the single model that models the user preferences, illustrating the importance of the item relationship modelling. Modelling the item relationships is more helpful in analysing user preferences.

## Conclusion

In this paper, a new sequential recommendation method based on a self-attention mechanism is introduced. The model considers the short- and long-term intentions of the user, alongside the relationship between items to infer the user’s next action. It utilizes self-attention to understand the user’s short-term intentions from their most latest behavior and to model the item relationships. Experiments are conducted on both datasets, and the model proposed in this paper achieves optimal performance compared to some other baselines because both the long- and short-term preferences of users and the relationship between items are considered. The analysis indicates that our model accurately obtains the importance of the relationship between user behavior and items. In addition, it is effective to extend the self-attention to a sequence recommendation method.

In the future, more work tends to include more additional information to further improve the accuracy of the recommendation, such as studying the time information of the user’s evaluation of the item, and hopefully more knowledge will be investigated to enhance this model. This work is also applicable to other relevant sequence recommendation tasks.

## Supporting information

S1 Data Data used in this model. (RAR)
